# Pitting Corrosion of Hot-Dip Galvanized Coatings

**DOI:** 10.3390/ma13092031

**Published:** 2020-04-26

**Authors:** Agnieszka Królikowska, Leszek Komorowski, Pier Luigi Bonora

**Affiliations:** 1 Bridge Department, Road and Bridge Research Institute, 03-302 Warsaw, Poland; 2 Associazione Italiana Tecnici Industrie Vernici ed Affini (AITIVA), 29-122 Piacenza, Italy

**Keywords:** HDG coating, lead in HDG bath, silicon in steel, corrosion

## Abstract

Lead (Pb) addition to hot-dip galvanizing (HDG) baths affects the physical characteristics of zinc coatings and is also useful to protect kettles. The influence of lead additions on both corrosion rate and morphology as well as on structure of zinc coating is less investigated. In this paper, three different additions, (Pb = 0.4–0.8–1.2 w/w) were chosen for three series of steel substrates, plus references without lead. The three steels chosen as substrates contained silicon (Si) = 0.18, 0.028, 0.225 w/w, respectively. The experimental part included both macro- and micro-electrochemical measurements, weight loss vs. time plots, Glow Discharge Optical Emission Spectroscopy (GDOS) and SEM/EDX microanalysis of both surface and cross-section of samples. Lead concentration is responsible for evident bimetallic coupling in the surrounding of lead inclusion with consequent increased dissolution rate, chunk effect, and rougher surface morphology.

## 1. Introduction

Hot-dip galvanizing found the first application after an original idea of a chemist in 1742. The first industrial plant in Italy was built in Milan in 1883. Since the first industrial applications, it has appeared to be among the best methods to protect steel surfaces against the aggressivity of atmosphere; continuous improvements were applied to get the best results both for corrosion resistance and for aesthetics, leaving alone the economic aspects linked to the thickness of zinc layers, the time of permanence in the bath and its bath composition and temperature, and the quality of the steel.

The widening of its use enhances the need for a well-defined relationship between aesthetics and durability, since the former can vary from shiny silver to a dull matte grey finish depending upon steel composition, bath composition, bath temperature, bath shape, and mass of the object. This concern has caused many research works to be published with the aim of optimizing the main function parameters of the coating, namely morphology (for both aesthetic and durability reasons), corrosion rate, and consequent corrosion morphology as related to environmental impact.

Both thickness and composition of the coating will be mainly driven by the process parameters, namely both bath and steel substrate composition and bath immersion time, not forgetting those parameters more linked to skillful operations such as shape of objects, temperature, time of immersion [1,2,3,4].

**Figure 1:**
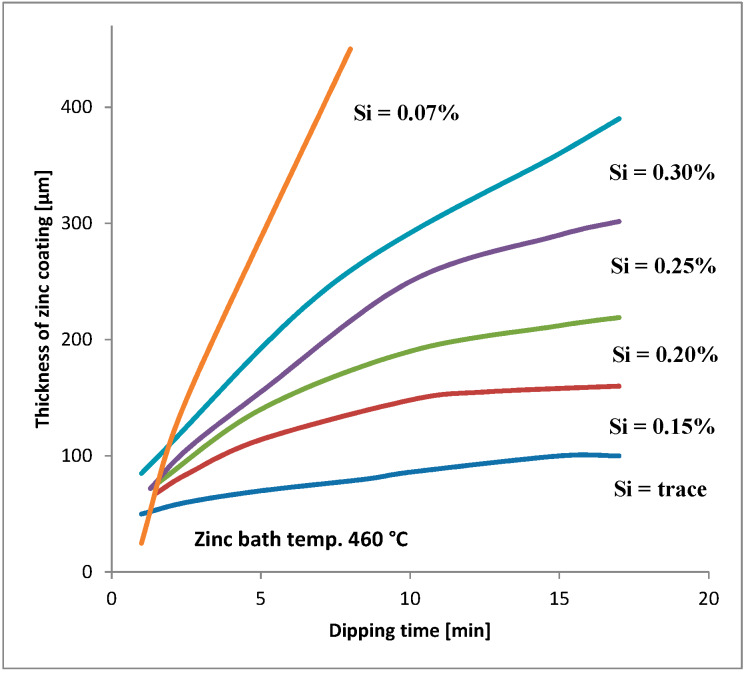
Relationship between dipping time and thickness of zinc coating in steels with different silicon contents [14].

Silicon is added to steels to remove oxygen. These steels are known as “killed steels”. The influence of the amount of silicon and phosphorus in steel on HDG coatings is widely described in the literature [5,6,7,8,9,10,11,12,13]. The silicon content should always be taken into consideration for steels that will be galvanized (see e.g., Figure 1). The silicon and phosphorus content is the basis for the following steel division in four groups: [2]

(1) low silicon < 0.03% (Si + P);
(2) from the Sandelin area 0.03%–0.12%;
(3) in the Sebist range of 0.12%–0.28%;
(4) high silicon 0.28%–0.60%.

In HDG, Al and Pb are added due to their influence on both thickness and brilliant appearance of the coating. Pb addition affects the physical characteristics of zinc, in particular both viscosity and surface tension [15].

The results are both a better wetting of steel tackle by molten zinc and due to an increased fluidity of the bath, an easier flow of zinc in excess from the surface of the coated object during the extraction from the tank and coatings thickness. Krepski [16] has shown that addition of 0.03%–1.2% weight Pb is decreasing zinc consumption up to 60%.

Pb additions are also useful to protect the tank and especially in the cyclic operation of extraction of mattes. In fact, they have a higher specific weight to the zinc and tend to be deposited in contact with the bottom of the tub. In the presence of a deposit of molten Pb lying on the bottom of the tank, the mattes will float. In this way, a gap is created between mattes and tub, which enables the appropriate buckets of slip, by performing the operation of their removal without the risk of hitting and damaging the bottom of the tank [17].

The standard ISO 14713-1:2019 [18] shows that HDG coatings should protect steel up to 10–20 years in the corrosive atmosphere C5 with the thickness of 85 µm. It was observed in facts that pitting corrosion sometimes appears already after 2–3 years both on roads and in urban infrastructures both in Poland and in Czech Republic [19,20].

The influence of Pb additions on both corrosion rate and morphology as well as on structure of zinc coating are less investigated and the available papers show controversial results [21,22,23,24,25,26,27,28,29,30,31,32].

They vary from concluding of no influence (Schulz and Thiele [2]) to an excessive influence on growth of zinc crystallites and their dendritic structure, therefore modifying both texture and appearance and affecting also positively corrosion protection [22]. Changes of crystals orientation and their influence on corrosion protection have been investigated by Changa and Shina [23] and in other works [24,25,26]. No effect on corrosion was observed after 20 years of tests in the field of the zinc coating with lead contents of 0.0055%, 0.049%, and 0.84% [27]. The same results are reported for percentages of 0.5% and 0.68% [28]. The results presented by Vala [29] show that addition of lead to zinc in galvanizing bath promotes the formation of large, smooth surface spangles providing good sacrificial ability. He concluded, by a comparison of calculated corrosion rates, that the optimum lead content requirement in the bath is 1.0 % to 1.5%. Data available in the literature are therefore dispersed most probably in reason of chunk effects [32,33] and bimetallic corrosion and the reliability this last statement [29] needs further confirmation.

In this paper, we intend to investigate the effects of lead on zinc layers grown on three steel substrates with different silicon content.

## 2. Materials and Methods

### 2.1. Samples Preparation

Hot-dip galvanizing coatings were prepared under laboratory conditions on the three steels:

(1) low silicon (≤0.03% w/w Si),
(2) low Sebist range (0.18% w/w Si),
(3) high Sebist range (0.225% w/w Si).

Their chemical composition was determined via the Spark OES method using a Magellan Q8 Bruker device. (Table 1).

The steel samples were degreased and rinsed in dichloroethane, etched for 20 min in an 18% HCl solution, then rinsed in demineralized water and immersed in Tegoflux 60 flux for approximately 5 min. After drying the samples were kept in a dryer at 140 °C, then they were hot-dip galvanized at 450 ± 0.5 °C for 6 min in zinc bathes with addition of 0–0.4–0.8–1.2 w/w Pb.

The lower amount of lead was chosen based on the average values used in galvanizing plants, and the upper range is below the solubility limit of lead at 450 °C (1.6%). For every test, the number of samples tested is given. zinc coatings were produced at the Silesian University of Technology in Katowice, Poland.

### 2.2. Weight Loss Measurements on Samples Exposed in the Salt Chamber

Gravimetric tests for weight loss were performed for zinc coatings exposed in a salt chamber (Klimatest HKT 500) for 16, 49, 63, 91, and 117 days. Before weighing, zinc corrosion products were selectively dissolved in an aqueous solution of chromium trioxide (200 g/L) at 80 °C for about 1 min, and then rinsed in distilled water and dried. The results are presented as an average value of three different specimens.

### 2.3. Morphology of the Zinc Layers

The morphology of the zinc layers was studied by means of a JEOL 6010 LV Scanning Electronic Microscope, both on upper surface and in cross-section, also detecting its average thickness. Chemical analysis was carried out through EDX analyzer. The results are presented as a significant representation of three measurements on two different specimens.

### 2.4. GDOS Profile Spectra

Glow Discharge Optical Emission Spectroscopy (GDOS) has been employed to describe the chemical profile of the samples. The experimental set – up has been described in [34]. Two samples for each measurement were used and each one was scanned five times to get an average reliable result.

### 2.5. Potentiodynamic Tests

The tests were performed in a three-electrode system (SCE reference, platinum counter-electrode and test sample as working electrode) in the potential range from −1.3 V SCE to −0.4 V SCE. A scanning speed of 0.2 mV/s was used. The IVIUM Stat set was used for electrochemical tests.

Every test was repeated three times for every sample of Table 2. The potential of the samples was tested before making polarization curves.

The tested surface area was the same every time. The tests were carried out in a 3% sodium chloride solution. Each time the potential was stabilized for 300 s.

### 2.6. Local Electrochemical Measurements

**Figure 2:**
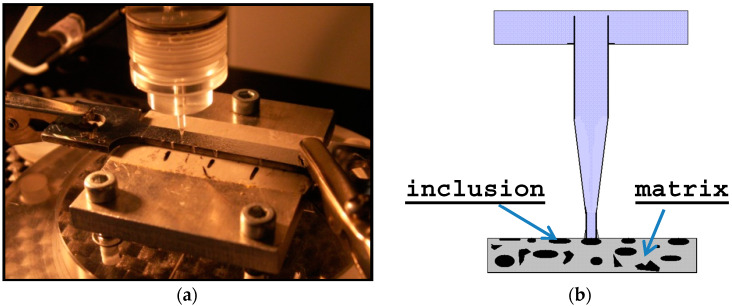
(**a**) Experimental set-up for micro-electrochemical analysis. (**b**) Measurement schematic diagram.

Local electrochemical behavior of samples was investigated using the technique of the local microcells. This technique allows analyzing the corrosion behavior of the material in the microscale [35,36,37,38]. The experimental set-up of the system is shown in Figure 2.

High resolution enables local electrochemical measurements in micro areas and electrochemical characterization of the behavior of the individual phases of metallic or intermetallic both inclusions and precipitates.

An 80-micrometer diameter micro-capillary was used. The micro-capillary is mounted in an electrochemical cell with platinum counter-electrode and Ag/AgCl electrode as a reference. Both cell and micro-capillary tube were filled with electrolyte (in this case 0.1 M NaCl). The microcell apparatus was homemade and the potentiostat an Autolab PGSlat-30. A scanning speed of 1.0 mV/s was used. The electrochemical cell is placed on the focus of one lens in the optical microscope, which ensures both the precise location of the micro-capillary and a measurement in a selected place on the surface of the working electrode, i.e., the sample (Figure 2)**.** The end of the capillary is covered with Silicone, which creates a seal, ensuring good contact of the micro-capillary with the surface of the tested sample and prevents leakage of electrolyte. Seeing that the measurements relate to very small areas and therefore low currents (on the order of nA, -pA), the whole system was placed in a Faraday cage. It fulfills the task of a screen protecting against external electromagnetic fields that might affect measurements.

Reliable measurements were possible only on samples with lead inclusions sized over 50 µm. The best result was obtained with A3 sample (1.2% Pb on low silicon steel). Tests were carried out at AGH in Krakow, Poland.

## 3. Results

Tests were made on the samples described in Table 2. The tests shown in the “Materials and Methods” part involved multiples of 12 different samples relevant to three different steel types in four different types of bath.

Table 2 includes also thickness of the coatings. It is evident that zinc coatings are thinner on steel substrate with 0 % of Si when compared to thickness of coatings on 0.18% Si steel. The higher percentage of Si (0.228%) again reduces the zinc coating thickness.

**Figure 3:**
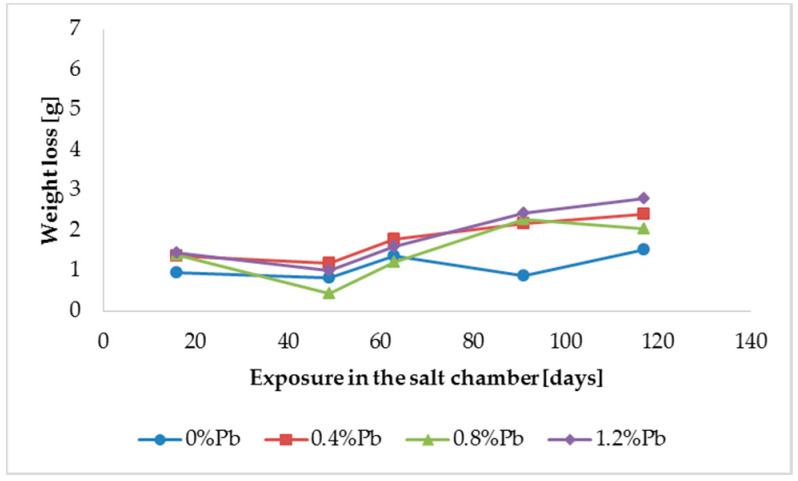
Weight loss for low silicon steel in the 117 days period in salt spray chamber.

**Figure 4:**
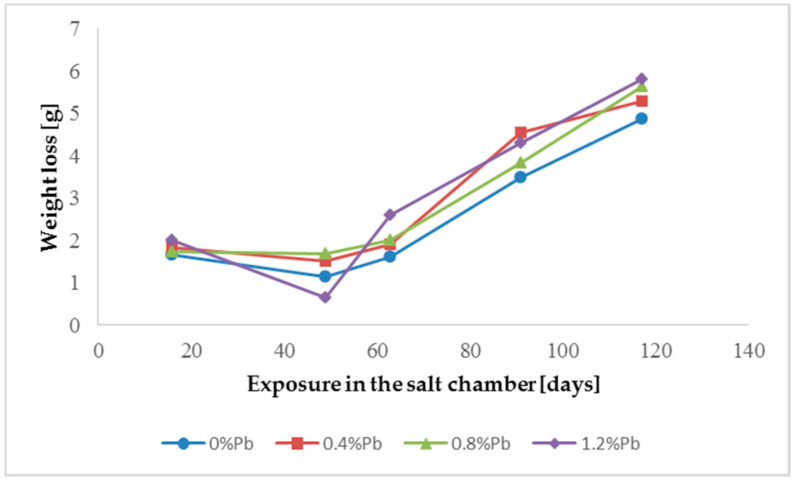
Weight loss for low Sebist range steel in the 117 days period in salt spray chamber.

**Figure 5:**
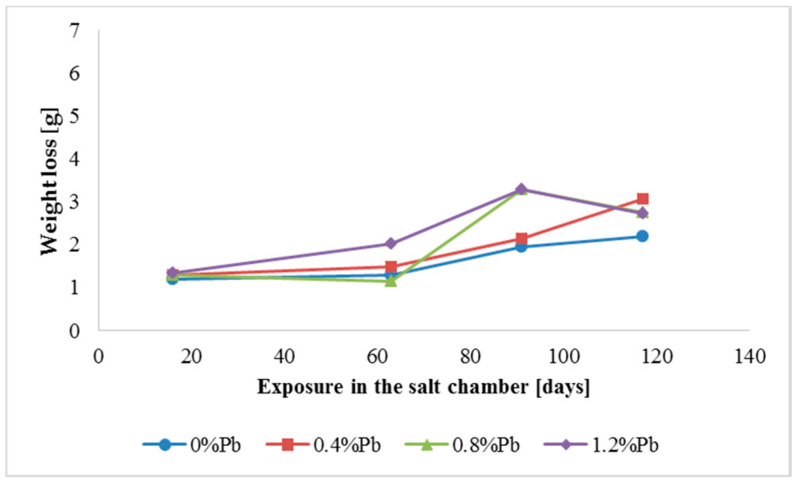
Weight loss for high Sebist range steel in the 117 days period in salt spray chamber.

The results of weight loss of the samples up to 117 days in salt spray chamber are displayed in Figure 3, Figure 4 and Figure 5.

Weight loss was the highest for low Sebist range steel. The two other steels showed similar weight losses.

**Figure 6:**
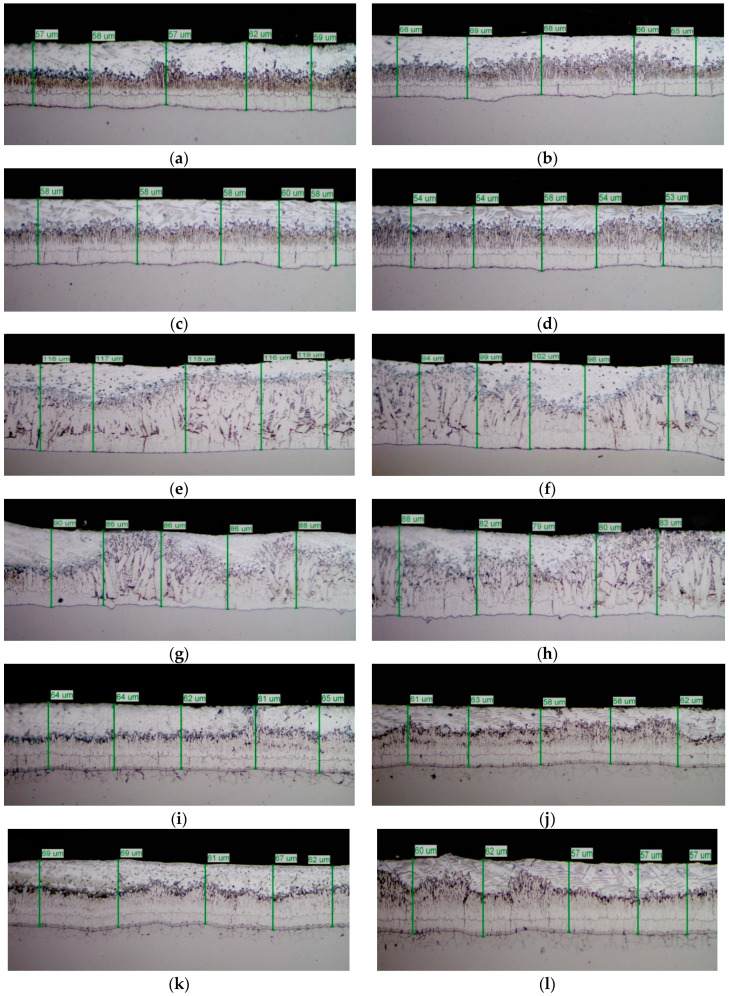
Phase distribution and thickness of HDG coatings. (**a**) A0, (**b**) A1, (**c**) A2, (**d**) A3, (**e**) B0, (**f**) B1, (**g**) B2, (**h**) B3, (**i**) C0, (**g**) C1, (**k**) C2, (**l**) C3.

**Figure 7:**
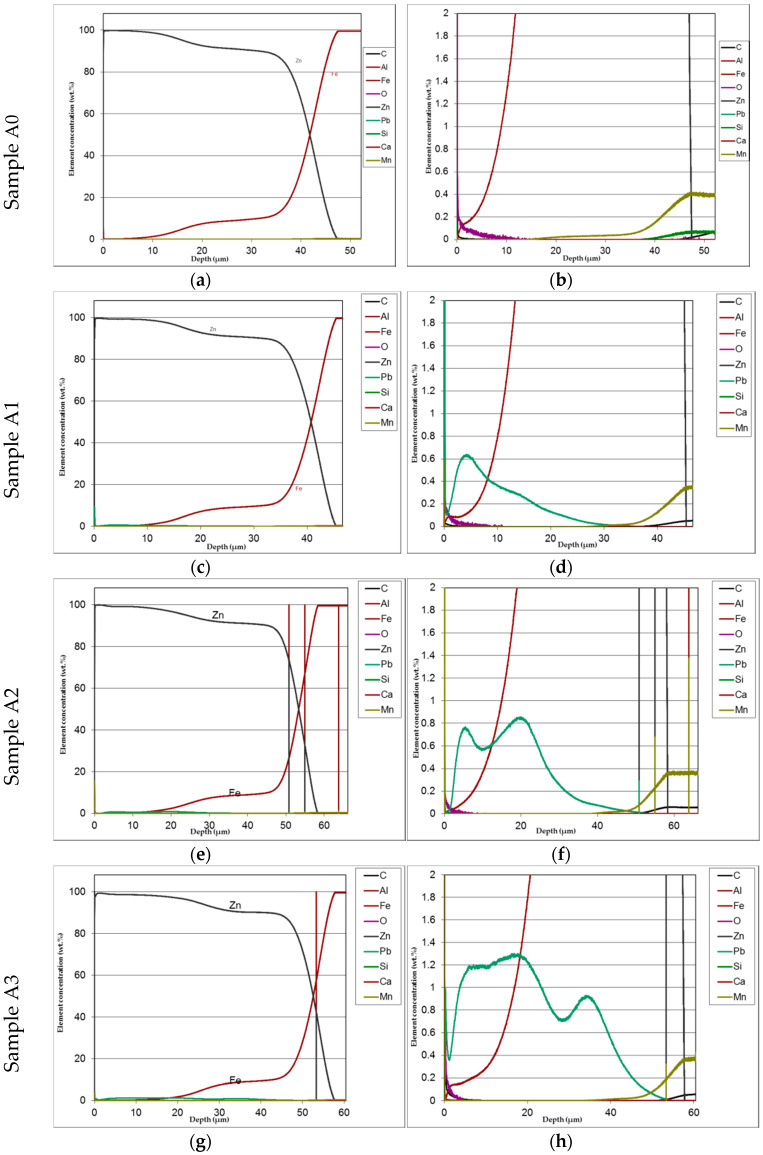
Example of GDOS profiles showing distribution of Fe and Zn along with coating cross-section for low silicon steel with and without lead addition (**b**,**d**,**f**,**h** show enlarged details of figures **a**,**c**,**e**,**g**).

The mirror finished cross-section of the samples was observed by SEM analysis (Figure 6) and submitted to GDOS in order to get a chemical profile along with the coating depth **(**Figure 7) before salt spray exposure. Table 3 summarizes the whole set of results including the approximate thickness evaluation of cross-section of coatings by both GDOS and SEM, including the evaluation of the extension of γ, δ, **ξ**, η, phases [1,2,3,16] by GDOS.

Both the low silicon steel (A0–A3) and high Sebist range steel (C0–C3) show well distinguished sequence of the phases. B–series shows more disordered view. Thickness values are displayed on top of every cross-section.

The distribution of phases in the coatings as well as of both iron and added lead are clearly displayed in the GDOS profiles as it can be seen, e.g., in Figure 7 relevant to low silicon steel.

**Figure 8:**
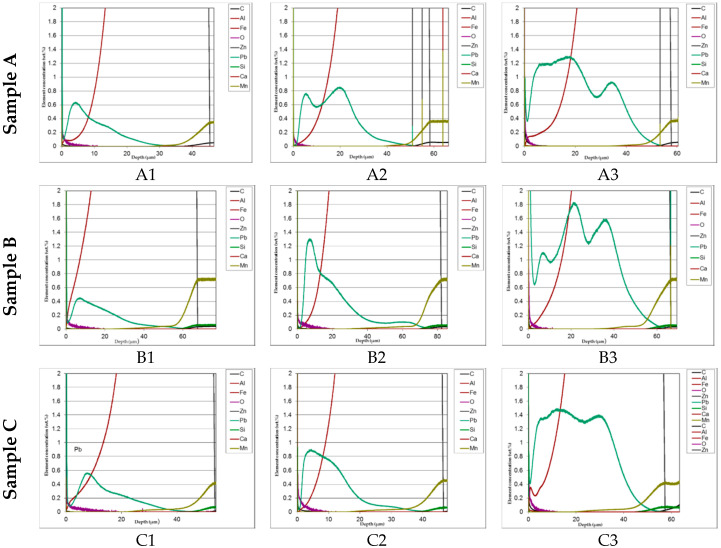
Comparison of the lead distribution showed on the GDOS dependently on steel type and amount of lead addition.

In Figure 8 comparison of the lead distribution showed on the GDOS dependently on steel type and amount of lead addition is shown.

The distribution of phases in the coatings as well as of both iron and added lead are clearly displayed in the GDOS profiles. High concentration of lead prevailingly accumulated towards the upper part of the layer. This effect is less evident at the highest lead content (1.2%). Table 3 summaries the phases thickness evaluations.

**Figure 9:**
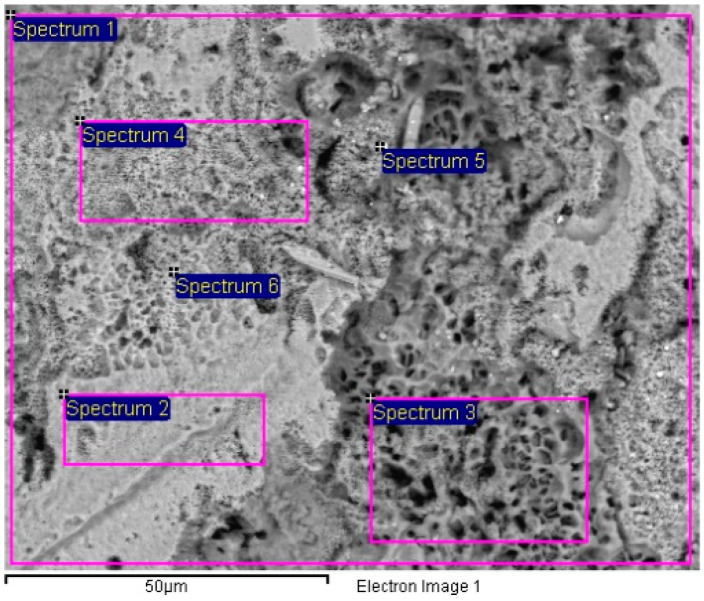
Corroded surface after 117 salt spray days exposure of Sample B2 showing the locations of EDX spectra listed in Table 4.

The corroded surface of samples after salt spray chamber was analyzed by SEM and EDX spectra were obtained in various locations relevant to the effect of Pb inclusions on surface morphology. An example representative of the obtained results is shown in Figure 9 relevant to sample B2 showing the locations of EDX spectra listed in Table 4.

An example of the effect of lead addition and consequent inclusion of lead in form of droplets on surface morphology is shown in Figure 9. Table 4 shows the differences of lead distribution on the tested surface because of the bimetallic corrosion.

**Figure 10:**
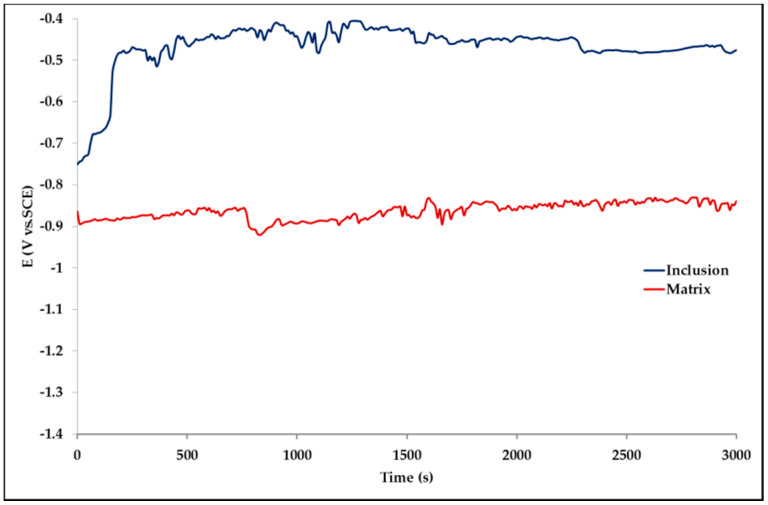
Corrosion potential measured on a Pb inclusion (**blue line**) and on a Pb-free Zn area (**red line**).

**Figure 11:**
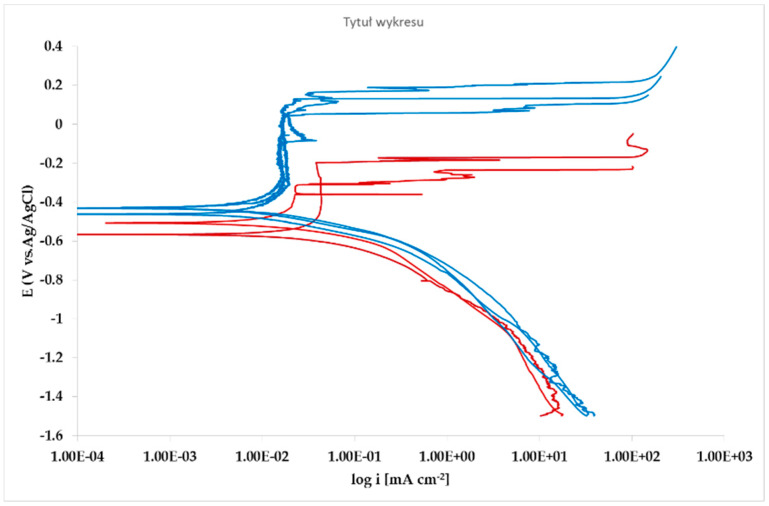
Microcell potentiodynamic E/log i plots on matrix (**red lines**). and on Pb inclusion (**blue lines**).

The effect of Pb inclusions has been further investigated by means of local micro-electrochemical analysis, which has shown evidence of the difference in both corrosion potential (Figure 10) and polarization behavior (Figure 11) between the Pb inclusion and the surrounding Zn matrix.

**Figure 12:**
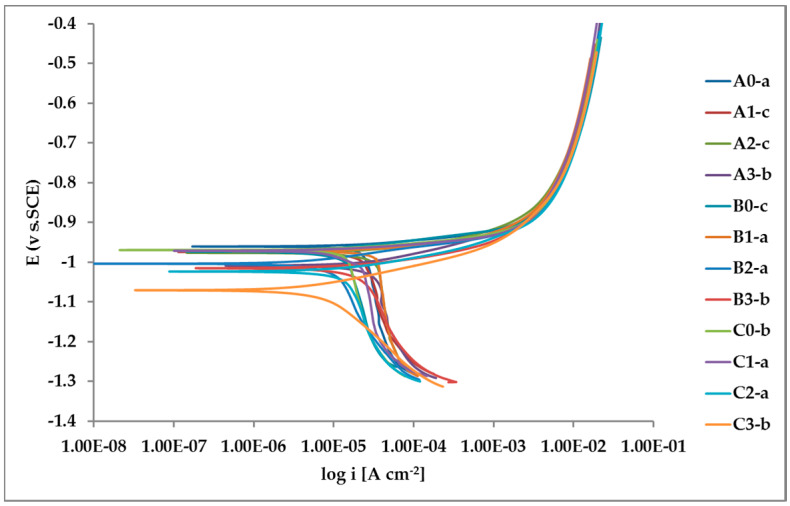
Potentiodynamic polarization curves for the whole set of samples.

The whole set of potentiodynamic curves carried out is shown in Figure 12.

The random distribution of the Pb inclusions accounts for the establishment of a mixed potential value which is in continuous evolution during the action of the local microcells. It follows that the average corrosion rate is evaluable only through the long-term weight loss, while the instantaneous corrosion rate is not giving any reasonably plottable value of corrosion rate through the calculation of polarization resistance.

Nevertheless, the E/log i plots shown in Figure 12 allow the argument that the rate-determining mechanism is linked to Zn dissolution, while the cathodic reactions depend on the instant conditions of the coupling with Pb inclusions.

## 4. Discussion

The aim of the research was to investigate the appearance of corroded areas with both aesthetic and functional effects on HDG structures on road infrastructure constructions in Poland. The analysis of extracted samples showed that the surface degradation was accompanied by the presence of lead inclusions. The study was directed to the three possible structural steels the composition of which might affect both shape and thickness of HDG, as a function of Si content, centered on low silicon and Sebist one. The Pb content span was chosen in order to obtain a single phase bath, usually used in practice, taking in account that at the normally used bath temperature of 450 °C the solubility limit is about 1.6 w/w %. At room temperature Pb is practically insoluble in zinc. It is distributed in the upper layers of the coating in form of randomly distributed droplets but for the highest concentration where more uniform distribution of droplets is observed, as both EDX and GDOS showed. Such a morphology is suitable to produce, due to the high distance between zinc and lead in the galvanic series, a consequent number of galvanic microcells producing an increase of zinc dissolution rate. This event is reflected by the results of the weight loss measurements.

HDG layers on steel are aimed to provide a stable, uniform surface aspect and a durable corrosion resistance in harsh environments. HDG is carried out in many different factories with a variety of bath formulations and on steels of different both structure and composition. Table 2 provides a matrix of the most common possible combinations of both lead additions to bath and silicon content in steel. Mainly weight loss (proportional to actual corrosion rate) and consequent surface morphology were interrelated as a function of layers structure, composition, and thickness. Figure 3, Figure 4, Figure 5, Figure 6, Figure 7 and Figure 8 together with Table 3 and Table 4 summarize the experimental results. It appears that zinc coatings grown on A- and C-samples show a clear and well distinguished sequence of phases, while on B-samples the **ξ** phase is more disordered with presence of crystals of different both shape and composition. The A- and C-samples show similar weight loss while for B-samples weight loss is double (about 2–3 g after 117 days compared to 5–6 g). The weight losses for all baths without lead were lower as compared to the alloyed ones. Up to 92 days for all steels the weight loss was proportional to the lead amount. Between 92 and 117 days this trend changed for C2 and C3 samples and for A2 samples.

Morphology of corroded samples mirrors the complexness of both structure and composition of the surface layers, including the presence of elements differing as for electronegativity. Fe is more evenly distributed due to higher solubility in Zn, while Pb is present as an archipelago of inclusions. The aggressive environment of salt spray causes many so-called “chunk effects”, i.e., removal of intact Pb particles due to bimetallic corrosion of the surrounding anodic Zn matrix. After 92 days, the removal of many Pb droplets allows the remaining zinc surface to be less affected by bimetallic corrosion with consequent lower weight loss.

After 48 days the weight loss is lower, probably because all corrosion products present at the surface of the samples were completely dissolved.

The cross-section SEM micrographs (Figure 6) show clearly that on samples A0, A1, A2, A3 the distribution of the phases along with the growth of zinc is pretty ordered, with a hardly perceivable thinning of the η layer and a progressive thinning of the whole layer along with increasing Pb content. On samples B0, B1, B2, B3 the η layer is intrinsically thinner (see sample B0 in comparison, e.g., with A0) and the Pb content worsens this phenomenon so that **ξ** phase is partly in contact with environment. Again, Pb tends to produce thinner coatings. On high Si steels (Samples C0, C1, C2, C3) Pb additions show no influence on both thickness and phase distribution. The analysis of GDOS spectra allows for an empirical evaluation of the HDG phases (Table 3). B samples give obviously thicker coatings, while Pb develops wider η phases, but extensively polluted by Pb particles.

Corrosion morphology is also affecting zinc dissolution rate as well as the aesthetics of the structures. From the EDX analysis (Table 4) of the corroded sample B2 shown in Figure 11 the complex both morphology and composition of the surface is displayed. The composition of the whole sample is reflected in the data of spectrum 1, while spectra 5 and 6 point out the composition of oxidized Pb inclusions. The comparison of spectra 2 and 3, both showing no Pb, reflects the relative inertness of an area where Pb inclusions were absent as compared with the areas relevant to spectra 3 and 5, where a dense population of inclusions accelerated local corrosion of the anodic surrounding zinc, with consequent chunk effect and detachment of the inclusions. Consequently, a mismatching follows between weight loss and corrosion current and hence corrosion rate. The micro-electrochemical measurements are a clear evidence of such situation. They account for the complexity of surface composition. As an example, the scattered electrochemical behavior is observed in different locations on the same sample. When the measurements are carried out on the Pb inclusion the corrosion potential is about 500 mV nobler than the value observed on zinc (Figure 10), while breakdown potential is about 300 mV nobler (Figure 11). The presence of very active bimetallic coupling is clearly detected. The analysis of potentiodynamic plots puts in clear evidence that the anodic branches are hardly distinguishable from each other. The complex mechanism of depolarization as well as of inhibition of both branches of the corrosion process leads to the conclusion that corrosion rate is mainly the result of zinc dissolution in NaCl solution. The random distribution of the Pb inclusions accounts for the establishment of mixed potential values which are in continuous evolution during the action of the local microcells. It follows that the average corrosion rate is evaluable through the long-term weight loss, while the instantaneous corrosion rate is not giving any reasonably plot table value of polarization resistance.

High lead additions are harmful for both durability and reliability of HDGcoatings. On site observations have shown that the effects are much more extended in occluded surfaces. Given the highly scattered results present in the literature of the topic, further investigations of the different environments as well as of the geometry of the structures are necessary.

Possible replacements with Bismuth (for both durability and ecology reasons) are studied at 10 times lower concentrations than lead, taking account of both size and distribution of Bi inclusions [39,40].

## Figures and Tables

**Table 1:** The composition of the steels used as substrates for HDG (w/w).

Steel	C	Mn	Si	P	S	Cr	Ni	Mo	Al	Cu	Ti	Fe
A	0.119	0.411	0.028	0.0151	0.0148	0.037	0.021	0.002	0.0349	0.037	0.0115	99.27
B	0.08	0.69	0.18	0.013	0.01	0.03	0.02	0.003	0.048	0.07	0.001	98.85
C	0.310	0.635	0.225	0.0108	0.0048	0.134	0.093	0.017	0.0245	0.286	0.0013	98.05

**Table 2:** Identification of samples and thickness (µm) of HDG (SEM).

% w/w Pb	Sample Number/Zn Coatings Thickness (µm)		
	0.00 % w/w Si Low Silicon Steel	0.18 % w/w Si Low Sebist Range Steel	0.225 % w/w Si High Sebist Range Steel
0.0	A0/68	B0/115	C0/64
0.4	A1/60	B1/100	C1/60
0.8	A2/58	B2/85	C2/60
1.2	A3/55	B3/80	C3/60

**Table 3:** Approximate cross-section thickness of coatings evaluated by both GDOS and SEM, including evaluation of extension of γ + δ, **ξ**, η phases by GDOS.

Sample Number	w/w % Si	w/w % Pb	Thickness of γ + δ Phases (µm)	Thickness of ξ Phase (µm)	Thickness of η Phase (µm)	Total Thickness Evaluated by GDOS (µm)	Total Thickness Evaluated by SEM (µm)
A0	0	0	15	23	10	48	68
B0	0.18	0	20	60	−	80	115
C0	0.225	0	10	24	8	42	64
A1	0	0.4	10	23	12	45	60
B1	0.18	0.4	10	55	−	65	100
C1	0.225	0.4	10	25	18	53	60
A2	0	0.8	11	29	18	58	58
B2	0.18	0.8	17	50	15	82	85
C2	0.225	0.8	11	26	10	47	60
A3	0	1.2	11	26	20	57	55
B3	0.18	1.2	13	33	20	66	80
C3	0.225	1.2	14	29	14	57	60

**Table 4:** EDX analysis of locations shown in SEM micrograph of Figure 9 (w/w %).

Spectrum	O (w/w %)	Fe (w/w %)	Zn (w/w %)	Pb (w/w %)	Total (w/w %)
Spectrum 1	10.51	0.65	87.77	1.07	100.00
Spectrum 2	5.20	0	94.80	0	100.00
Spectrum 3	18.39	2.53	79.07	0	100.00
Spectrum 4	6.74	0	92.11	1.15	100.00
Spectrum 5	5.66	0.45	17.94	75.96	100.00
Spectrum 6	7.31	0	23.62	69.07	100.00
